# Multi-omics and immunogenomics analysis revealed PFKFB3 as a targetable hallmark and mediates sunitinib resistance in papillary renal cell carcinoma: in silico study with laboratory verification

**DOI:** 10.1186/s40001-024-01808-5

**Published:** 2024-04-15

**Authors:** Zhongwen Lu, Yongsheng Pan, Songbo Wang, Jiajin Wu, Chenkui Miao, Zengjun Wang

**Affiliations:** 1 Department of Urology, The First Affiliated Hospital of Nanjing Medical University, No.300 Guangzhou Road, Nanjing, 210029 China; 2 Department of Urology, The Second Affiliated Hospital of Nantong University, Nantong, China

**Keywords:** Papillary renal cell carcinoma, Glycolysis-Immune Risk Signature, Single-cell RNA-seq, Immune infiltration, Progression, Sunitinib resistance

## Abstract

Glycolysis-related metabolic reprogramming is a central hallmark of human cancers, especially in renal cell carcinoma. However, the regulatory function of glycolytic signature in papillary RCC has not been well elucidated. In the present study, the glycolysis-immune predictive signature was constructed and validated using WGCNA, glycolysis-immune clustering analysis. PPI network of DEGs was constructed and visualized. Functional enrichments and patients’ overall survival were analyzed. QRT-PCR experiments were performed to detect hub genes’ expression and distribution, siRNA technology was used to silence targeted genes; cell proliferation and migration assays were applied to evaluate the biological function. Glucose concentration, lactate secretion, and ATP production were measured. Glycolysis-Immune Related Prognostic Index (GIRPI) was constructed and combined analyzed with single-cell RNA-seq. High-GIRPI signature predicted significantly poorer outcomes and relevant clinical features of pRCC patients. Moreover, GIRPI also participated in several pathways, which affected tumor immune microenvironment and provided potential therapeutic strategy. As a key glycolysis regulator, PFKFB3 could promote renal cancer cell proliferation and migration in vitro. Blocking of PFKFB3 by selective inhibitor PFK-015 or glycolytic inhibitor 2-DG significantly restrained renal cancer cells’ neoplastic potential. PFK-015 and sunitinib could synergistically inhibit pRCC cells proliferation. Glycolysis-Immune Risk Signature is closely associated with pRCC prognosis, progression, immune infiltration, and therapeutic response. PFKFB3 may serve as a pivotal glycolysis regulator and mediates Sunitinib resistance in pRCC patients.

**Supplementary Information:**

The online version contains supplementary material available at 10.1186/s40001-024-01808-5.

## Introduction

Renal cell carcinoma (RCC) is one of the fatal neoplasms in the genitourinary system, with an estimated 76,080 new cases and 13,780 deaths in the United States, 2021 [1]. Accounting for 70% of all RCC, clear cell RCC (ccRCC) is the most commonly occurring subtype, followed by infrequent subtypes papillary RCC (pRCC) and chromophobe RCC (chRCC) [2]. PRCC occupies the second most common type of RCC clinically and demonstrates an indolent behavior clinically. Due to a considerable disparity in the incidence rate of ccRCC, pRCC catches less notice and the molecular characteristics of pRCC are elusive. However, advanced pRCC preferentially exerts metastatic potential and evolves into more lethal disease than ccRCC [3]. Therefore, it is an urgent need to discover novel and targetable biomarkers that can override diagnostic, therapeutic, and survival challenges of pRCC patients.

Metabolic reprogramming is considered as a hallmark of human cancers. Among which, renal cancer is an ideal metabolic disease model, as a result of mutated genes relating to metabolic events [4, 5]. Multifarious metabolic events have been reported in renal tumorigenesis, including glycolysis, TCA cycle, glutamine metabolism, and ATP production [6–9]. Accordingly, glycolysis serves as an essential approach for cancer cells’ energy capture and proliferation maintenance. Especially, even with sufficient oxygen supply, cancer cells can uniquely convert glucose into lactate. This kind of aerobic glycolysis is also called Warburg effect, supporting high rates of tumor cell proliferation and metastasis [9]. Renal cell carcinoma (RCC) is essentially a metabolic disease characterized by a reprogramming of energetic metabolism [10–13], including glycolysis [5, 14, 15], mitochondrial bioenergetics [16], and lipid metabolism [17, 18]. To date, emerging glycolytic-related genes have been identified and studied in kidney ccRCC, but rare is known in pRCC.

Nowadays, tumor immunotherapy played an increasingly important role in clinical treatment of RCC patients. Immune checkpoint inhibitors (ICI) can enhance the antitumor activity and inhibit immune escape [19, 20]. Combined immunotherapeutic agents’ strategies in recent years has revolutionized the treatment of patients with renal cell carcinoma, such as anti-PD1, anti-PD-L1, and anti-CTLA-4 [21, 22]. Nevertheless, most patients did not benefit from immune checkpoint blocking, let alone sustained disease control [23]. Consequently, the underlying mechanism driving resistance needs to be further explored.

In this study, we extracted a set of glucose metabolism-related genes and performed comprehensive analyses to elucidate tumor immune infiltration, clinical relevance, and therapeutic targets of pRCC using bioinformatic methodology. PFKFB3, as a vital regulator of glycolysis, was finally screened out as a promising glycolytic biomarker in pRCC. PFKFB3-specific inhibitors PFK-015 and sunitinib could synergistically inhibit pRCC cell proliferation.

## Materials and methods

### Data extraction and preparation

We extracted eleven glycose metabolic-related gene sets (HALLMARK_GLYCOLYSIS, REACTOME_GLYCOLYSIS, REACTOME_GLUCOSE_METABOLISM, CUI_GLUCOSE_DEPRIVATION, WP_GLYCOLYSIS_AND_GLUCONEOGENESIS, KEGG_GLYCOLYSIS_GLUCONEOGENESIS, GOBP_GLUCOSE_CATABOLIC_PROCESS, GOBP_GLUCOSE_METABOLIC_PROCESS, GOBP_POSITIVE_REGULATION_OF_GLUCOSE_METABOLIC_PROCESS, GOBP_REGULATION_OF_GLUCOSE_METABOLIC_PROCESS, PID_INSULIN_GLUCOSE_PATHWAY) from the Molecular Signatures Database v7.4 [24, 25] (MSigDB; http://www.gsea-msigdb.org/gsea/msigdb/). After removing duplicate genes, the entire glycose-metabolic-related gene set consisted of 520 genes (Table 1). Next, the normalized RNA-seq profiles (FPKM), matched clinical characteristics and survival information of 289 pRCC samples and 32 normal control samples from The Cancer Genome Atlas (TCGA, https://portal.gdc.cancer.gov) and GTEx database were downloaded.

**Table 1:** Eleven glycose metabolic-related gene sets screened from MSigDB

Gene-sets	Description	Genes
HALLMARK_GLYCOLYSIS	Genes encoding proteins involved in glycolysis and gluconeogenesis	200
REACTOME_GLYCOLYSIS	Glycolysis	72
REACTOME_GLUCOSE_METABOLISM	Glucose metabolism	92
CUI_GLUCOSE_DEPRIVATION	Representative genes up-regulated in MiaPaCa2 cells under glucose-deprived conditions	61
WP_GLYCOLYSIS_AND_GLUCONEOGENESIS	Glycolysis and Gluconeogenesis	45
KEGG_GLYCOLYSIS_GLUCONEOGENESIS	Glycolysis / Gluconeogenesis	62
GOBP_GLUCOSE_CATABOLIC_PROCESS	The chemical reactions and pathways resulting in the breakdown of glucose, the aldohexose gluco-hexose	36
GOBP_GLUCOSE_METABOLIC_PROCESS	The chemical reactions and pathways involving glucose, the aldohexose gluco-hexose. d-glucose is dextrorotatory and is sometimes known as dextrose; it is an important source of energy for living organisms and is found free as well as combined in homo- and hetero-oligosaccharides and polysaccharides	210
GOBP_POSITIVE_REGULATION_OF_GLUCOSE_METABOLIC_PROCESS	Any process that increases the rate, frequency or extent of glucose metabolism. Glucose metabolic processes are the chemical reactions and pathways involving glucose, the aldohexose gluco-hexose	41
GOBP_REGULATION_OF_GLUCOSE_METABOLIC_PROCESS	Any process that modulates the rate, frequency or extent of glucose metabolism. Glucose metabolic processes are the chemical reactions and pathways involving glucose, the aldohexose gluco-hexose	119
PID_INSULIN_GLUCOSE_PATHWAY	Insulin-mediated glucose transport	26

### Weighted gene correlation network analysis (WGCNA)

To identify the significant genes associated with the sample subtypes (tumor/normal) for the pRCC patients, we constructed a co-expression network using R package “WGCNA”. We conducted a co-expression analysis of pair-wise genes using Pearson correlation coefficients. PickSoftThreshold function was used to calculate the value of β (a soft threshold power parameter) for increase the similarity matrix and achieve a scale-free co-expression network. Associated genes were clustered based on dissimilarity of the unsigned topological overlap matrix (TOM). Finally, we constructed a tree diagram using hierarchical clustering and calculated the correlation between the module eigengenes (MEs) and the clinical traits used to screen the MEs related to the sample subtypes for the pRCC samples.

### Unsupervised consensus cluster analysis

Consensus clustering analysis was employed to stratify the pRCC samples into distinct subgroups using the “ConsensusClusterPlus” R package [26]. The curve of cumulative distribution function (CDF) and area under the CDF were used to choose the best k-value for the optimal cluster number.

### DEGs analysis and PPI network construction

The “limma” R algorithm was used to calculate differentially expressed genes (DEGs) between distinct groups [15]. Genes with false discovery rate (FDR) adjusted *P* < 0.05 and | logFC (fold-change) |> 1 were considered as DEGs.

PPI network was constructed using the STRING (http://string-db.org) database, which is an online biological database that could help to uncover critical regulatory genes [27]. Cytoscape software and Metascape website was further applied to visualize the PPI network [28].

### Construction of a predictive model and validation

Based on candidate DEGs we identified, univariate cox analysis, multivariate cox analysis and LASSO regression were performed sequentially to screen out possible prognostic WGCNA-glycosis-immune-related genes. Glycolysis-Immune Related Prognostic Index (GIRPI) was calculated with the following formula:

$${\text{GIRPI}}\, = \,\Sigma {\text{coef}}*{\text{Exp}}({\text{genes}})$$

On the basis of the median score, we divided all pRCC patients into low-GIRPI and high-GIRPI groups. Kaplan–Meier curve was applied for demonstrating pRCC patients’ survival status. To evaluate the predictive ability of these risk model, we further analyzed the receiver operating characteristic (ROC) curve and the area under the ROC curve (AUC) by R package “survivalROC”. Nomogram was established according to the patient’s overall survival comprising independent prognostic factors. The calibration curves were constructed to evaluate the consistency of prediction between actual survival observation and predicted clinical outcome in 1, 3 and 5 year. The sensitivity and specificity of the nomogram was measured by ROC curves and area under curve (AUC).

### Functional enrichment and pathways annotation

Gene ontology (GO) enrichment analysis including biological process (BP), cellular components (CC), molecular function (MF), together with Kyoto Encyclopedia of Genes and Genomes (KEGG) pathway annotation were applied using the R package “clusterProfiler” [29]. A q-value < 0.05 was considered statistically significant.

### Evaluating extent of immune cell infiltration abundance in tumor immune microenvironment

To exhibit the comprehensive landscape of immune cell infiltration in different subgroups, we conducted single-sample gene-set enrichment analysis (ssGSEA) and currently acknowledged algorithms, including XCELL [30, 31], TIMER [32, 33], QUANTISEQ [34, 35], MCPCOUNT [36], EPIC [37], CIBERSORT [33, 38] and CIBERSORT-ABS [39] to estimate the subpopulations of immunity infiltration scores. Differences between two risk groups were analyzed by the Wilcoxon signed-rank test and the results were obtained according to *P*-value < 0.05. Subsequently, we used correlation analysis when exploring the relationship between the risk score and immune infiltrated cells. Immune checkpoint genes were obtained from Auslander et al. [40].

### Prediction of response to potential chemotherapy drugs

R package “pRRophetic” and GDSC website were used to estimate half maximal inhibitory concentration (IC50) of common chemotherapeutic agent [41]. The difference between the high-GIRPI and low-GIRPI groups was compared by Wilcoxon signed-rank test. Furthermore, another database Connectivity Map (CMap; https://portals.broadinstitute.org/cmap) was used to discover potential small molecular compounds or drugs which may reverse or induce the biological states based on the differently expressed genes [42]. The enrichment score from − 1 to 0 suggested that the gene expression in high-risk group might be suppressed by these candidate drugs for pRCC patients. Finally, these candidate drugs’ 3D structure tomographs were obtained from PubChem (https://pubchem.ncbi.nlm.nih.gov), respectively, a public database of small molecules and their biological characteristics.

### Single-cell RNA sequence processing

Firstly, GSE152938 and GSE131685 were downloaded from GEO database, and the R package “Seurat” was used to process data (https://satijalab.org/seurat/) [43–46]. Two datasets containing one pRCC sample and four adjacent normal kidney samples were merged and integrated with “Harmony” algorithm [47]. After filtrating with the criteria of > 20% mitochondria-related genes, or less than 500 genes expressed or less than 1000 counts detected, we finally detected 30,797 cells for further analysis. After quality control, we normalized the data and rescaled all the RNAs. Next, respective reduction of cell clustering was performed and cell cluster was obtained through the Uniform Manifold Approximation and Projection (UMAP) method. Finally, we used common marker genes to get the cell type for cell population annotation.

### Cell culture and qRT-PCR

The renal cancer cell lines (786-O, 769-P, ACHN, Caki-1, Caki-2) and human renal tubular epithelial cell line (HK-2) were purchased from the Type Culture Collection of the Chinese Academy of Sciences (Shanghai, China) and cultured in RPMI 1640 (786-O, 769-P); McCoy’s 5A (Caki-1, Caki-2); DMEM (ACHN) and DMEM/F12 (HK-2) (Gibco, Thermo Fisher Scientific, USA) containing 10% fetal bovine serum and 1% penicillin/streptomycin (Gibco, Thermo Fisher Scientific, USA). PFK15 (PFK-015; Selleck, China), 2-Deoxy-d-glucose (2-DG; Selleck, China), Sunitinib (SU11248) malate (Sunitinib; Selleck, China), and dimethylsulfoxide (DMSO; Sigma–Aldrich, USA) were also used. Cells were transfected with control siRNA and siRNA-PFKFB3 using Lipofectamine 3000 (Invitrogen, Thermo Fisher Scientific, USA). QRT-PCR and Western blot assays were used to evaluate the efficiency of siRNA interference. Total RNA was isolated using Trizol (Invitrogen, Thermo Fisher Scientific, USA). HiScript III All-in-one RT SuperMix (Vazyme, China) was used for cDNA synthesis. qRT-PCR was performed with SYBR qPCR Master Mix (Vazyme, China) using StepOne Plus (Applied Biosystems, USA) and LightCycler 480 PCR instrument (Roche Diagnostics, Switzerland) according to the manufacturer’s instructions. The primers and siRNA Oligo used are listed in Additional file 1: Table S1.

### Cell proliferation and colony formation assays

Pretreated ACHN cells were counted and seeded into a 96-well plate at a density of 1.0 × 10^3^ cells/well. Cell proliferation was measured after 24 h, 48 h, 72 h, and 96 h using the CCK-8 Cell Counting Kit (Vazyme, China). The absorbance was measured at 450 nm with a microplate reader following incubation at 37 °C for 1 h according to the manufacturer’s protocols.

For the colony formation assay, pretreated cells were seeded into 6-well plates (1000 cells/well). The cells were incubated for 10 days. Colonies were fixed in 4% paraformaldehyde for 20 min, washed with PBS twice, and stained with 0.1% crystal violet for further analysis.

### Transwell cell migration assay

A total of 1.5 × 10^4^ cells pretreated cells were seeded into the 24-well Transwell upper chambers with serum-free medium for the migration assays. Medium containing 20% FBS was added to the bottom chamber. After incubation at 37 °C for 24 h, the cells were fixed in 4% paraformaldehyde for 20 min and stained with 0.1% crystal violet for 20 min. Cells were captured on a microscope in five randomly selected fields, and all of the experiments were repeated three times.

### Glucose concentration, lactate secretion, and ATP production measurement

The glucose concentrations assay, lactic acid production assay, and ATP detection assay kits (Jiancheng Bioengineering, Nanjing, China) were used according to the manufacturer’s protocol in renal cancer cell ACHN. Each level was normalized to the cell number.

### Statistical analysis

All analyses were performed with R version 4.1.1 (http://www.R-project.org) and corresponding packages. An independent t-test was used to compare continuous variables that exhibited normal distributions. The Wilcox test was used to compare the continuous variables that were not normally distributed. Kaplan–Meier survival analysis and Cox hazard regression model were employed to assess the overall survival, disease-specific survival, disease-free interval, and progression-free interval prognostic factors. All experiments were repeated at least three times. All statistical tests were two sided, and *P* < 0.05 was considered statistically significant.

## Results

In the current study, we constructed an effective Glycolysis-Immune Related Prognostic Index (GIRPI) to predict survival outcomes and performed comprehensive analyses to elucidate tumor immune infiltration, clinical relevance, and therapeutic targets of pRCC patients using bioinformatic methodology. Our findings revealed that PFKFB3, as a vital regulator of glycolysis, was a promising targetable glycolytic biomarker in pRCC treatment. PFKFB3-specific inhibitors PFK-015 and sunitinib could synergistically inhibit pRCC cell proliferation.

### Characteristic of immune infiltration landscape in pRCC subtypes

**Fig. 1:**
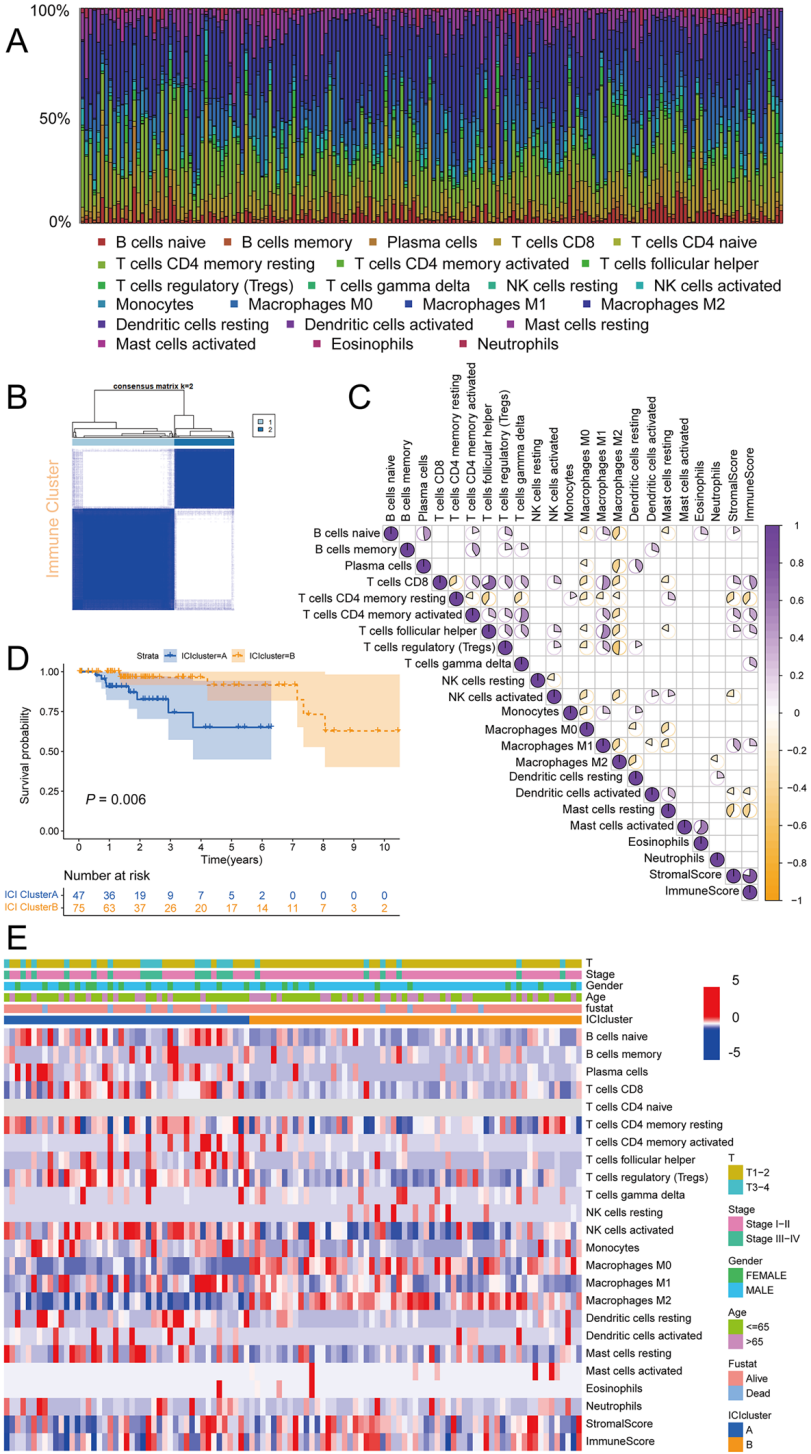
Characteristic of tumor microenvironment immune infiltration landscape in pRCC subtypes. **A** The abundance of each TME infiltrating cell subpopulations in pRCC samples. **B** Consensus clustering analysis to divide two distinct immune subtypes based on immune signatures estimated by ssGSEA score. **C** Spearman correlation analysis of different immune infiltration cells. **D** Survival analyses of the two immune cluster patterns based on pRCC patients. **E** The abundance of each TME infiltrating cell in ICI cluster A and B. *P < 0.05; **P < 0.01; ***P < 0.001. ns, not significant

We first investigated the landscape of 22 immune cell subpopulations infiltration in pRCC tissue using the CIBERSORT algorithm (Fig. 1A). Consequently, total pRCC samples were hierarchically clustered into two distinct immune subtypes based on immune signatures estimated by ssGSEA score (Fig. 1B, Additional file 1: Fig. S1E). Interestingly, we also found that the abundance ratios of some types of immune cells were correlated with other types (Fig. 1C). Moreover, we revealed the association between different immune cell subsets and pRCC clinical information including age, gender, stage, and T classification in a combined heatmap (Fig. 1E). The subtypes we defined as ICI cluster A and ICI cluster B had significant discrimination on immunogenicity. The majority of the immune scores, including B cells, Plasma cells, NK cells, Monocytes, Macrophages M0, Macrophages M2 and Dendritic cells, revealed significant differences among the subtypes as well (Additional file 1: Fig. S2A). Comparison of overall survival (OS) between two clusters showed significant differences (*P* = 0.006, Fig. 1D). Furthermore, we performed GSVA analysis between two ICI cluster and the result showed that ICI cluster A mainly related to Notch signaling pathway and cell cycle, while ICI cluster B enriched in sugar and nucleotide sugar metabolism, glycosaminoglycan degradation, other glycan degradation and PPAR signaling pathway (Additional file 1: Fig. S2B). Above results implied that the ICI cluster B subtype might have better responses to ICIs than cluster A subtypes, since previous studies demonstrate that tumor immune cell infiltration, HLA and immune checkpoints expression were positively associated with immunotherapeutic responsiveness.

### Two distinct subclasses mediated by glycolysis-related genes

**Fig. 2:**
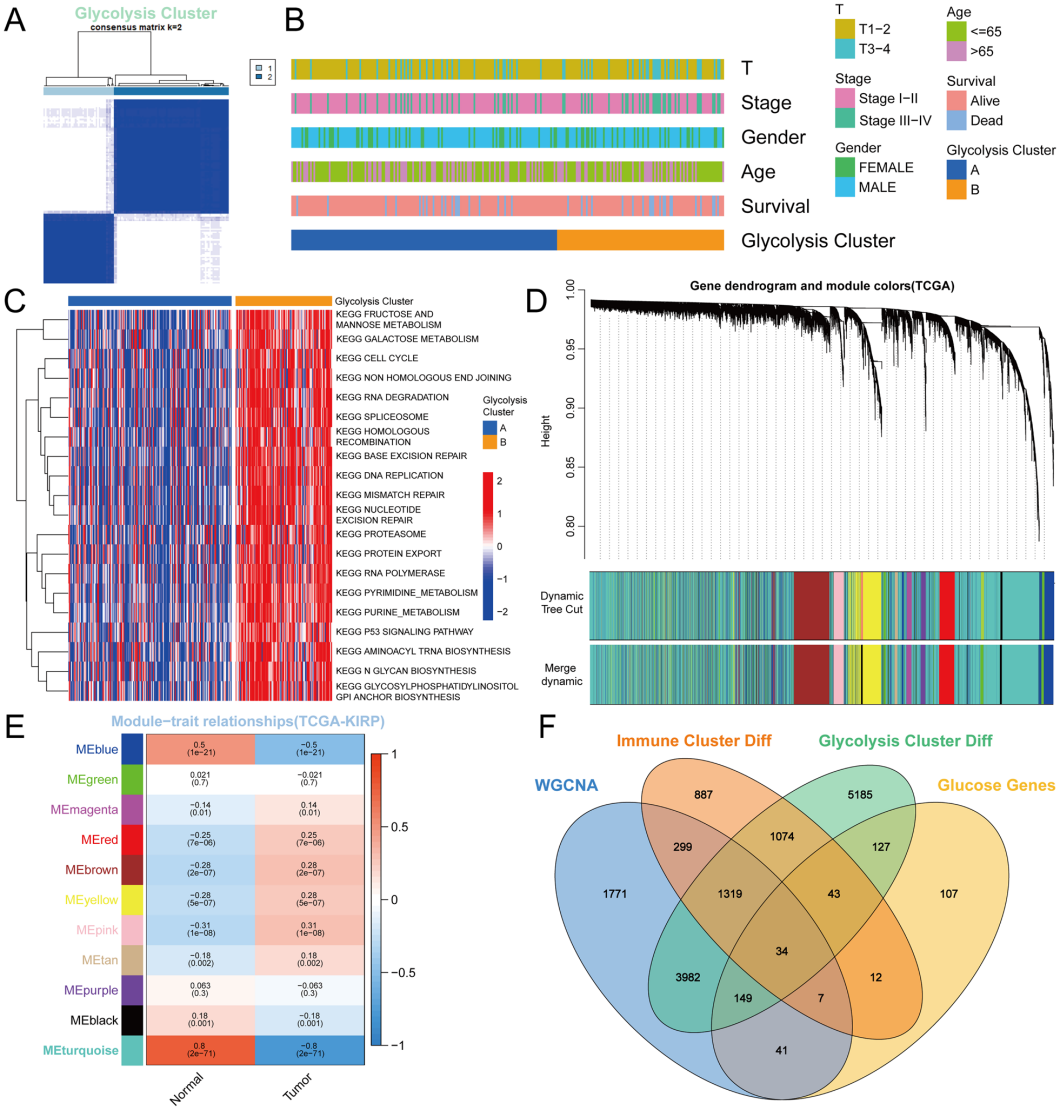
Subclass clustering mediated by glycolysis-related genes and WGCNA. **A** Unsupervised consensus cluster analysis to determine two distinct subclasses mediated by glycolysis-related genes. **B** The glycolysis clusters, T classification, tumor stage, gender, survival status and age were used as patient annotations. **C** GSVA enrichment analysis of two glycolysis clusters. **D** Cluster dendrogram of all gene’s distribution based on a dissimilarity measure in WGCNA network. **E** Heatmap of the correlation between module eigengenes (MEs) and sample characteristic (Normal/Tumor) of pRCC. Each module contained the correlation coefficient and displayed in different colors. **F** A Venn diagram showing WGCNA-glycolysis-immune-related overlapping DEGs

Based on the expression profiles of glycolysis-related genes, cluster analysis was performed to analyze the 289 pRCC samples from the TCGA database, and the comprehensive correlation coefficient was used to determine the optimal k-value as 2 (Fig. 2A, Additional file 1: Fig. S1F). Subsequently, two distinct subclasses were determined, ultimately dividing them into 2 groups: glycolysis clusters A and B. (Fig. 2B). And the results of KEGG pathway enrichment analysis showed that high-glycolysis cluster was significantly related to cell cycle, galactose metabolism, fructose and mannose metabolism, RNA degradation, and DNA replication pathway (Fig. 2C).

### WGCNA to filter significant modules and essential DEGs related to pRCC

First, we reanalyzed TCGA-KIRP RNA-seq data to determine DEGs between 289 tumor samples and 32 normal tissues. A volcano plot and heatmap showed the top 100 differentially expressed genes (Additional file 1: Fig. S1A-B). Next, we constructed a DEGs co-expression network to explore the functional key modules and genes related to pRCC oncological characteristics by WGCNA algorithm.

In this research, the soft-thresholding power was defined as two to achieve relatively balanced scale independence and connectivity network according to the scale-free topology criterion (Additional file 1: Fig. S1C). Here, eleven distinct modules with different colors were generated for further analysis. The correlation of candidate genes with tumor characteristics was calculated using the module-trait relationships method (Fig. 2D). Importantly, the turquoise module showed the strongest correlation with oncological characteristics (Fig. 2E). The corresponding correlation coefficient between module membership (MM) and Gene Significance (GS) was 0.9 (*P* < 1e-200), indicating the turquoise module was highly significantly associated with pRCC oncological characteristics (Additional file 1: Fig. S1D). Hence, a total of 7602 genes in the turquoise module were filtered out as key genes for subsequent research.

### WGCNA-glycolysis-immune-related overlapping DEGs in pRCC

According to the above WGCNA and consensus clustering analysis, expression profile comparison was further conducted between the “low-glycolysis & high- glycolysis” and “high & low immune responses to ICI” groups. After overlapping with glycolysis genes and WGCNA significant module genes, a total of 34 glycolysis-immune-related essential DEGs were identified. The Venn diagram demonstrated that there were 34 overlapping genes between the different groups (Fig. 2F).

### PPI network and function annotation for DEGs

**Fig. 3:**
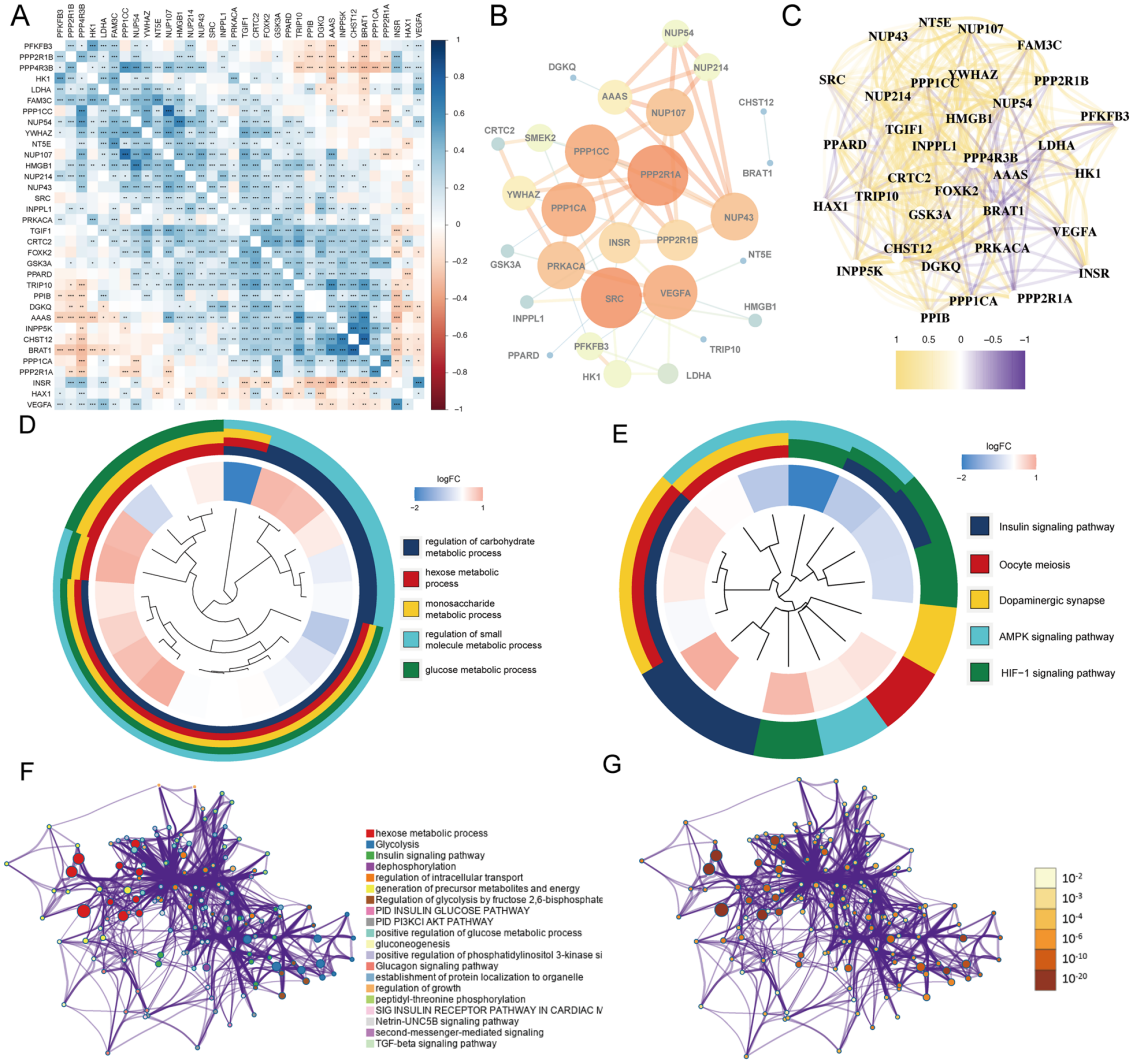
Interaction among 34 WGCNA-glycolysis-immune-related overlapping genes and functional enrichment. **A** Correlation analysis of 34 WGCNA-glycolysis-immune-related overlapping genes in the TCGA-KIRP. **B** The construction of PPI network by Cytoscape software. The thicker line represents higher connection strengths. **C** Correlation network at the transcriptional level by Spearman’s rank correlation analysis. **D** Circle diagram of Gene Ontology (GO) analysis for overlapping genes. **E** Circle diagram of Kyoto Encyclopedia of Genes and Genomes (KEGG) analysis for overlapping genes. **F** Network of enriched terms colored by cluster identity. **G** Network of enriched terms and genes colored by *P*-value

We investigated the expression correlation among these 34 genes and found that they share a high correlation in expression level (Fig. 3A). What’s more, we used STRING website and Cytoscape software to establish the PPI network and further visualize the interaction network. Some meaningful hub genes including PPP1CC, PPP2R1A, PPP1CA, SRC, VEGFA, and NUP43 were highlighted and shown in Fig. 3B, respectively [27]. Furthermore, we found that these genes were strongly correlated at the transcriptional level (Fig. 3C).

To discover biological functional and related pathways in DEGs based on WGCNA, glycolysis subtypes, and immune subtypes, we performed GO and KEGG analyses. As shown in Fig. 3D, the most significant GO terms were enriched in carbohydrate metabolic process, hexose metabolic process, and glucose metabolic process. For KEGG analysis, these 34 genes may strongly participate in Insulin signaling pathway, HIF-1 signaling pathway, and AMPK signaling pathway (Fig. 3E). In the meantime, Metascape tool was applied to construct PPI network and analyze functional enrichment, which was colored by different cluster subgroups (Fig. 3F) and *P*-values (Fig. 3G). The result confirmed that these 34 genes are primarily involved in glycolysis and other metabolic processes. Combined with the result shown in Fig. 3D–G, our study demonstrated that these DEGs were correlated mainly with the glucose metabolic process or immune-related pathways including AMPK pathway and TGF-β pathway in pRCC patients.

### Establishment of glycolysis-immune related prognostic index (GIRPI) and validation

From above analysis, a combination of 34 genes originated from the glycose metabolic-related genes, glycolysis subtypes, immune subtypes and WGCNA was analyzed through LASSO Cox Regression algorithm. Then, nine candidate genes (PPP4R3B, LDHA, PPIB, YWHAZ, NUP107, PFKFB3, FOXK2, CRTC2 and NUP43) were screened out. Meanwhile, univariate Cox regression was confirmed prognosis-related genes by the cutoff of *P* < 0.05 (Additional file 1: Fig. S3A). Finally, multivariate Cox analysis were conducted to filter out most powerful genes and construct a GIRPI related to OS (Additional file 1: Fig. S3B). GIRPI of pRCC patients was calculated according to the expression level of gene and regression coefficient as follows:

- GIRPI = 0.00506799913379297* (the expression level of LDHA) + 0.353700829182504* (the expression level of NUP107) + 0.0166719469192151* (the expression level of PFKFB3) + 0.15593066911614* (the expression level of CRTC2) + 0.256809447625349* (the expression level of NUP43) (Additional file 1: Figure S3C).

**Fig. 4:**
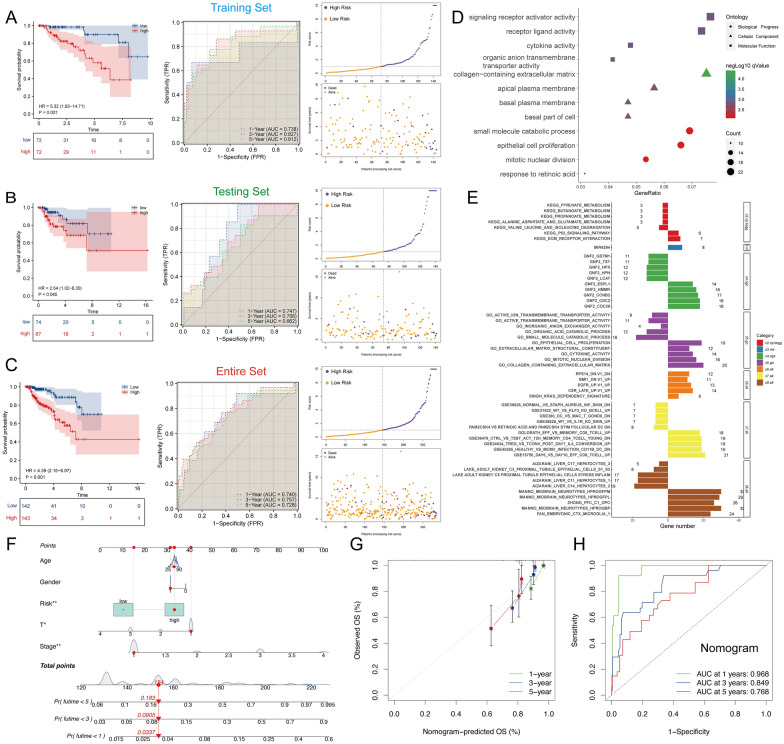
Establishment of a Glycolysis-Immune Related Prognostic Index (GIRPI) and function enrichment. **A**–**C** Kaplan–Meier survival analysis and ROC curves of high-GIRPI and low-GIRPI patients in training cohort (**A**); testing cohort (**B**) and entire cohort (**C**), along with the distribution of risk score and survival status. **D** GO and KEGG pathway enrichment analysis based on DEGs between high-GIRPI and low-GIRPI subgroups. **E** The result GSEA pathway annotation. **F** Construction of predictive nomogram. **G** Calibration curve of the nomogram at 1, 3, and 5 years. **H** Assessing prognostic performance of Nomogram by ROC curves

Next, we randomly allocated TCGA-KIRP samples into the training and testing sets to evaluate the predictive ability of the model, and patients in each set were separated into the high and low-risk groups on account of median risk score. Kaplan–Meier survival analysis and time-dependent ROC curves were utilized to estimate the prognostic signature. High-GIRPI group patients had significantly poorer outcomes in the training cohort (*P* < 0.001, Fig. 4A). The area under the curve (AUC) values in the training set were 0.761 at 1 year, 0.853 at 2 years, 0.833 at 3 years, 0.881 at 4 years, and 0.827 at 5 years. Coincidentally, the testing set and the entire set have consistent results with the training set, survival outcomes and prognosis of high-risk patients were worse compared to low-risk patients (Testing set: *P* = 0.038, Entire set: *P* < 0.001). The AUC values in the testing set were 0.747 at 1 year, 0.778 at 2 years, 0.705 at 3 years, 0.636 at 4 years, and 0.637 at 5 years, whereas the AUC values in the entire set were 0.740 at 1 year, 0.810 at 2 years, 0.758 at 3 years, 0.747 at 4 years and 0.721 at 5 years (Fig. 4B, C). The distribution of GIRPI and survival status are presented in Fig. 4A–C. Additionally, the expression levels and clinical characteristics of five prognostic genes in the high-risk group and the low-risk group were shown by heatmap (Additional file 1: Figure S3D). Above research confirmed that GIRPI shows a good predictive capability for clinical outcomes of pRCC patients.

### Kaplan–Meier survival and genetic alteration landscape of five signature genes in pRCC patients

We further analyzed the association between GIRPI five-component genes and the survival state (OS, DFS) of pRCC patients. Kaplan–Meier survival curves showed that the OS and DSS of pRCC patients with higher expression of CRTC2, LDHA, NUP43, NUP107, and PFKFB3 were significantly shorter than those with lower expression (Additional file 1: Figure S4A-B, Fig. 7C). In conclusion, our results demonstrated that these five signature genes were potential prognostic biomarkers that can accurately predict survival outcomes. Genetic alteration information of five signature genes was explored by querying cBioPortal database to discover the potential influence of genetic alteration upon the corresponding gene expression [48] (Additional file 1: Fig. S5A). Among them, the mutation frequency of CRTC2 was the highest (1.7%), followed by PFKFB3 (1%). Using TIMER database, the copy number variation (CNV) of five signature genes, including deep deletion, arm-level deletion, diploid/normal, arm-level gain, and high amplification, significantly affected the infiltration levels of B cells, CD4 + T cells, CD8 + T cells, neutrophils, macrophages, and dendritic cells in pRCC [32, 49] (Additional file 1: Figure S5B).

### GSEA analysis revealed differences between high-GIRPI and low-GIRPI groups

To further elucidate the underline biological mechanisms between the high-GIRPI and low-GIRPI groups, we performed GO, KEGG, and GSEA enrichment. GO enrichment analysis revealed that high-GIRPI group is mainly involved in the signaling receptor activator activity, collagen-containing extracellular matrix, and small molecule catabolic process (Fig. 4D). In addition, KEGG pathways annotation results showed that high-GIRPI group was related to ECM receptor interaction and p53 signaling pathway (Fig. 4E). Above analysis revealed that molecular function was closely associated with the malignant properties of pRCC, especially proliferation.

### Predictive nomogram based on GIRPI and clinical features

To appraise the clinical application ability for pRCC patients’ overall survival prognosis, we integrated the GIRPI and other clinical characteristics to build a nomogram. GIRPI, age, gender, stage, and T classification were included in the nomogram (Fig. 4F). In addition, the corresponding calibration plots in 1, 3, and 5 years were also drawn (Fig. 4G). As shown in Fig. 4H, the AUC values of 1-, 3-, and 5-year OS predictions for the nomogram were 0.968, 0.849, and 0.768, respectively. In general, the above results show that the nomogram performed well at predicting overall survival in clinical pRCC patients (Fig. 4F–H).

### Correlation of GIRPI with tumor immune microenvironment

**Fig. 5:**
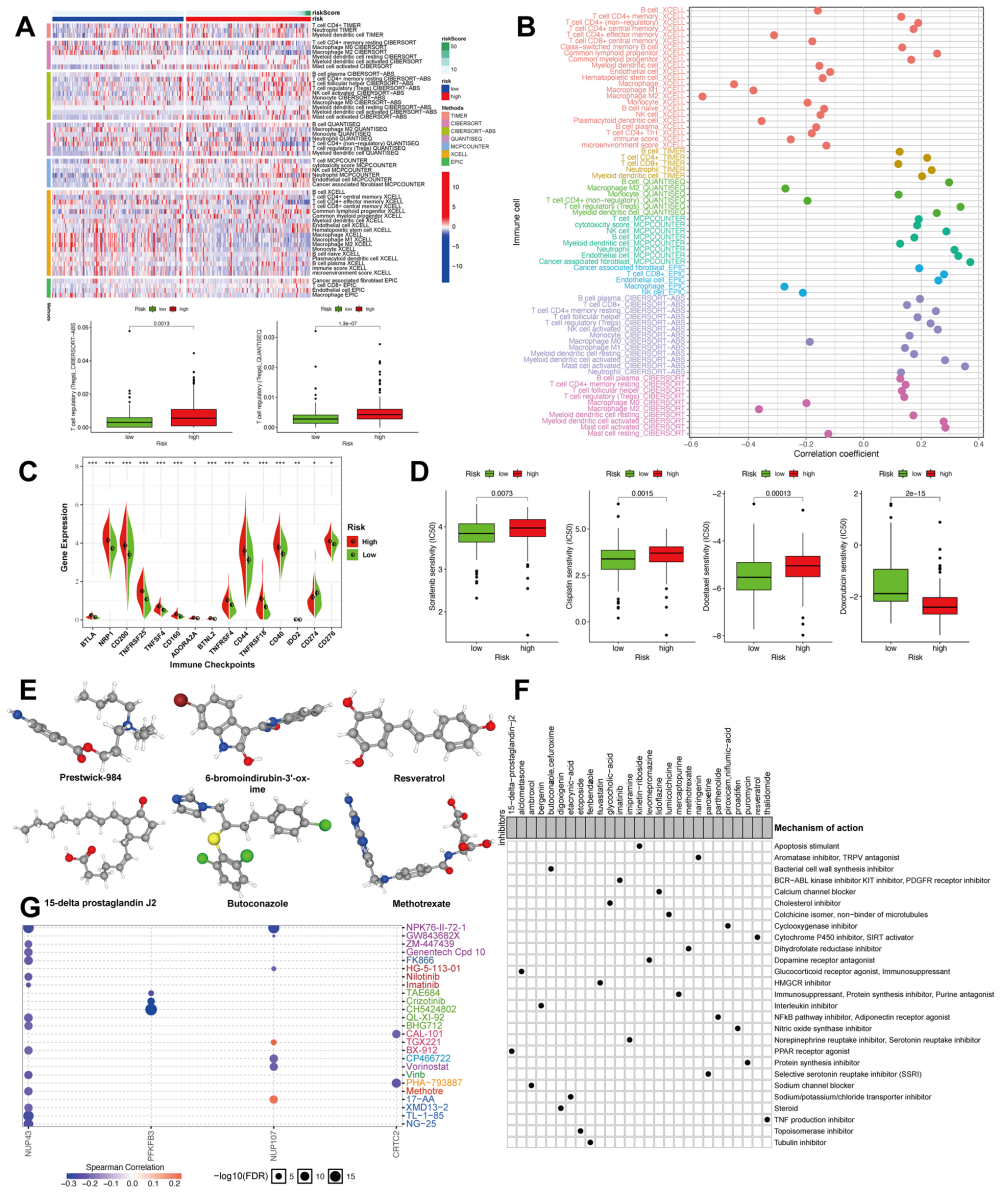
Correlation of GIRPI with tumor immune-infiltrating characteristics and screening out the potential therapeutic strategies for pRCC patients based on GIRPI. **A** The histogram exhibits the different immune cell fractions between high-risk and low-risk groups. **B** Spearman correlation between risk score and immune infiltration. **C** The histogram compared several major immune checkpoints gene expression between the high-risk group and low-risk group. **D** Sensitivity prediction of four commonly used chemotherapeutic drugs. **E** The 3D structure tomography of candidate small-molecule drugs targeting GIRPI signature (Prestwick-984, resveratrol, 15-delta prostaglandin J2, 6-bromoindirubin-3'-oxime, methotrexate, and butoconazole). **F** CMap mode-of-action (MoA) analysis of potential compounds. **G** Bubble plot of the correlation of NUP43, NUP107, CRTC2, and PFKFB3 in GDSC drug sensitivity database. The color from blue to red represents the correlation between mRNA expression and IC50. The bubble size positively correlates with the FDR significance

Considering the heterogeneity and complexity of the tumor immune microenvironment in pRCC patients, we carried out seven different algorithms, including XCELL [30, 31], TIMER [32, 33], QUANTISEQ [34, 35], MCPCOUNT [36], EPIC [37], CIBERSORT [33, 38] and CIBERSORT-ABS [39] algorithm to explore the composition of tumor-infiltrating immune cells between high-risk and low-risk groups (Fig. 5A, B). Compared with low-risk groups, we accordingly found that Cancer-associated fibroblast and T cell regulatory (Tregs) were enriched in high-risk groups’ TME (Fig. 5A). Furthermore, we compared several major immune checkpoints gene expression between the high-risk group and low-risk group and found that BTLA, NRP1, CD200, TNFRSF25, TNFSF4, CD160, ADORA2A, BTNL2, TNFRSF4, CD44, TNFRSF18, CD40, IDO2, CD274 and CD276 highly expressed in high-risk group (Fig. 5C).

### Potential therapeutic strategy for pRCC patients based on GIRPI

Notably, we compared the response to drugs (IC50) using the R package “pRRophetic” to further evaluate the influence of GIRPI on predicting drug therapy response [41]. Four common chemotherapy drugs showed a significant difference in chemotherapy drug sensitivity. Among them, patients in the low-GIRPI subgroup might be more sensitive to Sorafenib, Cisplatin, and Docetaxel, which means patients may benefit from these chemotherapeutic drugs (*P* < 0.01 for Sorafenib, Cisplatin, and Docetaxel). However, we found high-GIRPI subgroup exhibit a lower IC50 in doxorubicin, suggesting that these patients are better candidates for this treatment (Fig. 5D). Next, the CMap database (https://portals.broadinstitute.org/cmap) was conducted to screen out candidate small-molecule drugs showing therapeutic effects on pRCC. Based on differently expressed genes between high-GIRPI and low-GIRPI groups, six small-molecule drugs were finally selected (*P* < 0.05 and enrichment score < − 0.6) (Table 2). The 3D structure tomography of these small-molecule drugs (Prestwick-984, resveratrol, 15-delta prostaglandin J2, 6-bromoindirubin-3'-oxime, methotrexate, and butoconazole) was found in the PubChem (https://pubchem.ncbi.nlm.nih.gov), which may provide possible solutions for clinical treatment (Fig. 5E). CMap mode-of-action (MoA) analysis revealed 27 mechanisms of action shared by the above compounds [42] (Fig. 5F).

**Table 2:** Potential therapeutic small molecular compounds from connectivity map (CMap) website based on GIRPI

CMap name	Mean	N	Enrichment	*P*-value	Specificity	Percent non-null
Resveratrol	− 0.616	9	− 0.769	<0.00001	0.0093	88
15-Delta prostaglandin J2	− 0.327	15	− 0.627	<0.00001	0.0301	60
6-Bromoindirubin-3ʹ-oxime	− 0.445	7	− 0.81	0.00002	0	71
Methotrexate	− 0.414	8	− 0.717	0.00012	0.0069	62
Butoconazole	− 0.643	4	− 0.852	0.00092	0.0076	100
Prestwick-984	− 0.403	4	− 0.831	0.00147	0.0056	75
Puromycin	− 0.475	4	− 0.804	0.00284	0.0448	75
5707885	− 0.236	4	− 0.788	0.00406	0.0168	50
Bergenin	− 0.342	4	− 0.772	0.00547	0.0065	50

In the meantime, we performed a drug sensitivity analysis to evaluate the correlation between five model genes and clinical outcomes. Results indicated that NPK76-II-72-1, CH5424802, and Crizotinib were connected with drugs and small molecules in the GDSC database [50] (Fig. 5G).

### Single-cell RNA-seq revealed the distribution of GIRPI genes

**Fig. 6:**
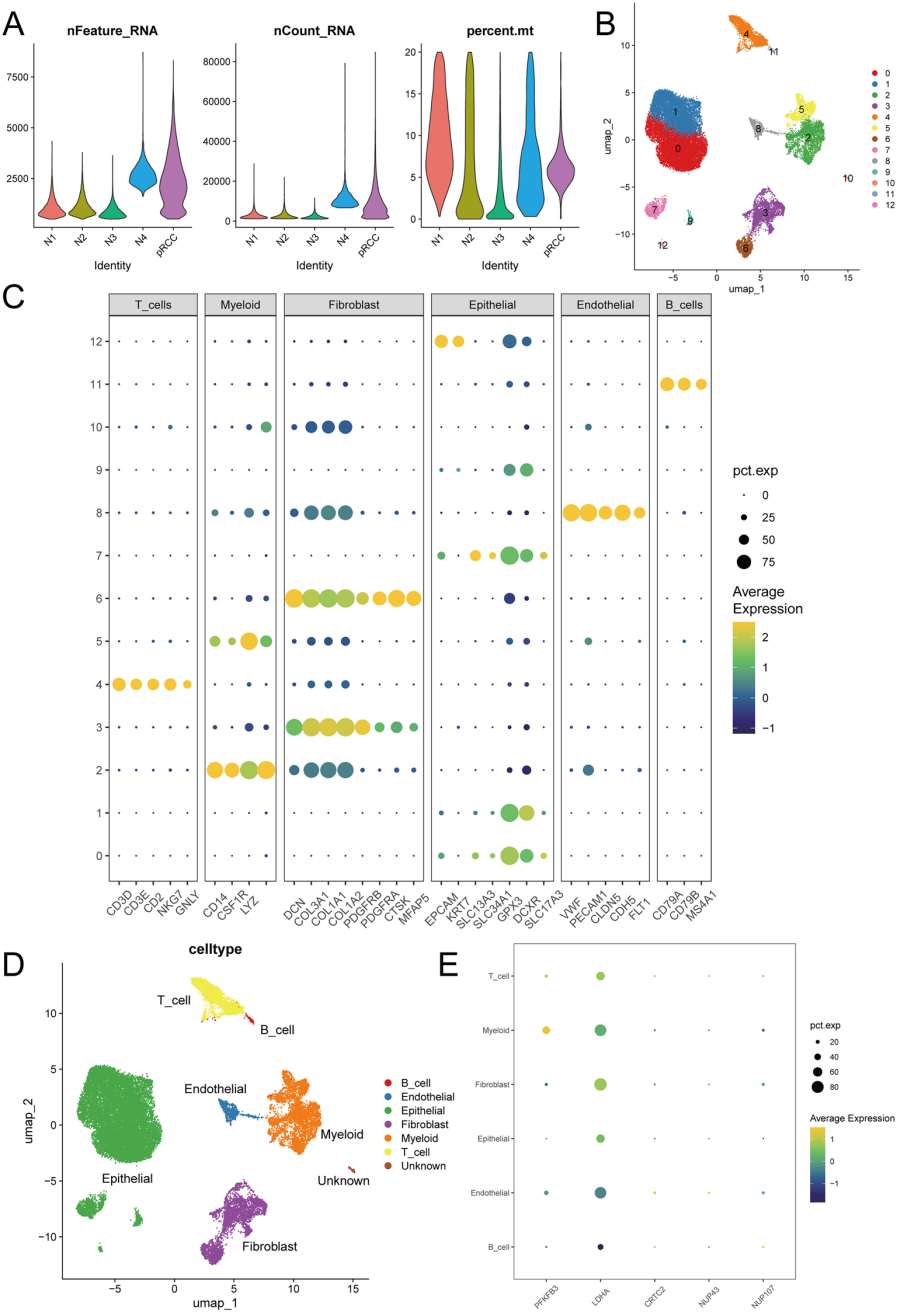
Single-cell RNA-seq revealed the distribution of GIRPI genes. **A**. Quality control of single-cell RNA-seq samples (Four normal kidney samples: N1-N4 and one pRCC sample). The number of gene expressions in each cell, the sum of gene expressions, and the percentage of mitochondrial genes were illustrated. **B** UMAP plot showing total 13 clusters. **C** The results of the cell cluster obtained by common cell marker gene. **D** UMAP plot showing six main cell types distribution in the integrated dataset. **E** Dot plot showing the expression levels and distribution of PFKFB3, LDHA, CRTC2, NUP43 and NUP107 in different cell based on ESCC scRNA-seq dataset

In addition to bulk-RNA sequencing, we studied the cellular composition of pRCC with single-cell RNA-seq datasets GSE152938 and GSE131685 [43, 46]. Firstly, we performed quality control to ensure the reliability of cells for the following analysis (Fig. 6A). Four normal kidney samples and one pRCC sample were analyzed, yielding 30,797 high-quality transcriptomes after quality control and filtering. After performing PCA and UMAP dimensionality reduction, unsupervised clustering in each compartment gave rise to a total of 13 clusters (Fig. 6B). Then, we used common marker genes to annotate different cluster cells. PRCC samples could mainly be divided into epithelial cells, endothelial cells, fibroblasts, myeloid cells, T cells, and B cells (Fig. 6C, D). Finally, we tried to explore the exact distribution of GIRPI genes in pRCC tissues. The graphs obtained by t-SNE algorithm revealed that expression of PFKFB3 was most concentrated in myeloid cells; LDHA was mainly distributed in fibroblasts, which indicates these genes play a vital role in glycolysis and immune (Fig. 6E and Additional file 1: Figure S6).

### Experiment verification of five WGCNA-glycolysis-immune-related genes

**Fig. 7:**
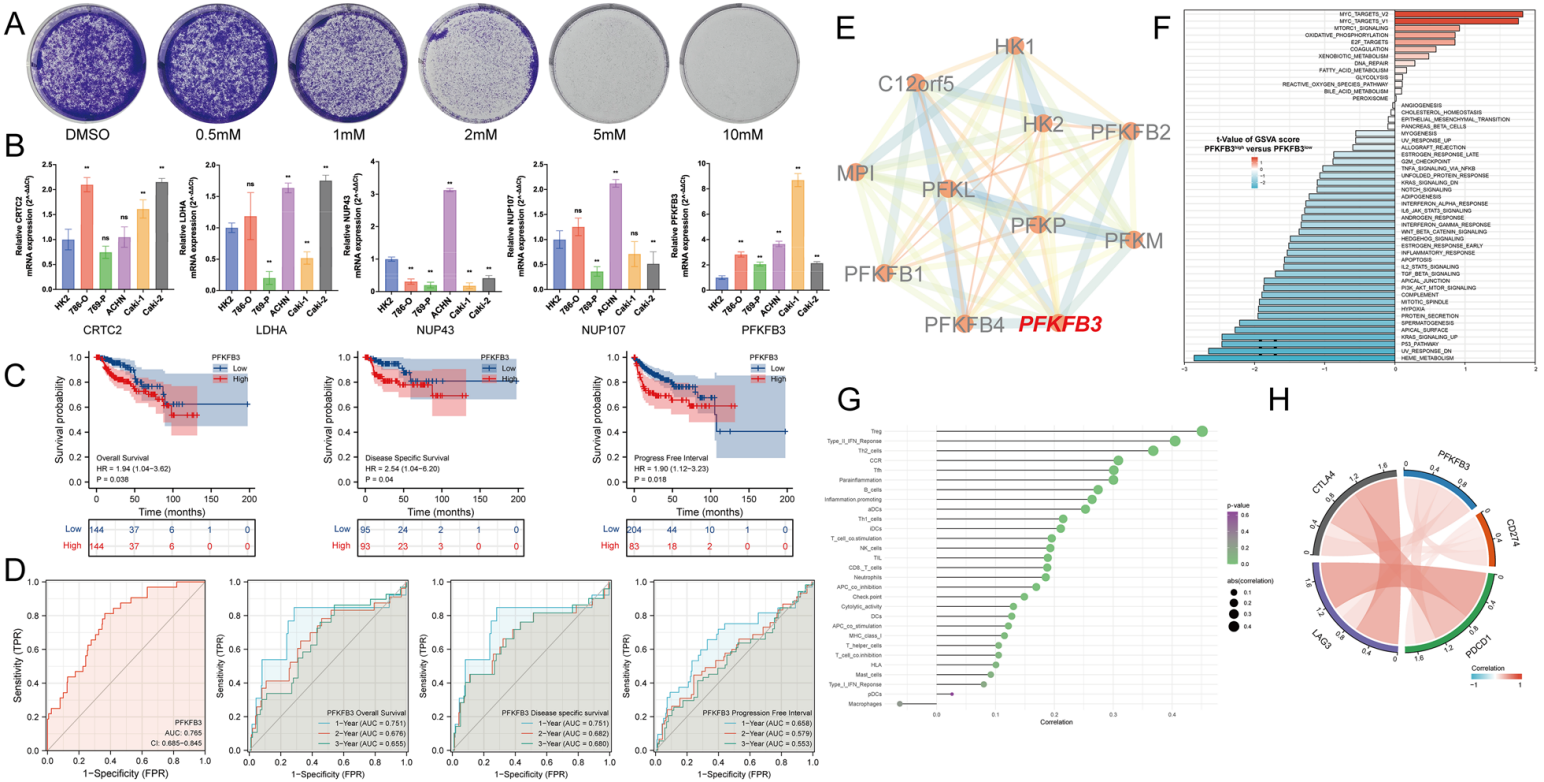
The association with clinical relevance and Immune infiltration of key GIRPI component gene PFKFB3. **A** Clone formation capability of ACHN cells exposed to different concentrations of 2-DG. **B** qRT-PCR analysis to verify the expression level of five GIRPI genes in RCC cell lines. **C** Kaplan–Meier survival of PFKFB3 in pRCC patients (OS, DSS, PFI). **D** The time-dependent ROC curves of PFKFB3. **E** PPI network of PFKFB3-related genes using STRING database. **F** GSVA analysis of potential mechanism involved in PFKFB3. **G** The underlying association between PFKFB3 and immune infiltration of cells. **H** The association between some immune microenvironment molecules and PFKFB3

To support tumor growth, high glycolysis rate is required to generate ATP to synthesize macromolecules required for cancer cell mass-replication during division and proliferation [51]. Therefore, we examined the potential influence of 2-DG (a glucose analog that acts as a competitive inhibitor of glucose metabolism) in pRCC cell proliferation using colony formation assays. 2-DG significantly suppressed the clone formation capability of ACHN cells at low concentrations (1 μM and 2 μM). At 5 μM and 10 μM concentrations, 2-DG almost entirely inhibited the clone formation of ACHN cells (Fig. 7A). These results confirmed that targeting glycolysis presents a promising strategy for pRCC cancer therapy.

Next, to verify the expression level of five WGCNA-glycolysis-immune-related genes in renal cell lines, we performed qRT-PCR experiments. Among them, PFKFB3 was generally upregulated in all renal tumor cell lines (786-O, 769-P, ACHN, Caki-1, and Caki-2). These findings were consistent with the results analyzed in TCGA dataset (Fig. 7B). Li et al. reported that blockage of glycolysis by targeting PFKFB3 suppresses head and neck squamous cell carcinoma growth and metastasis [52]. However, the role of PFKFB3 in pRCC patients has yet to be elucidated. Accordingly, we chose target gene PFKFB3 for further research.

### Clinical relevance and immune infiltration analysis of PFKFB3

To further investigate whether PFKFB3 was correlated with prognosis in pRCC patients, we used the Kaplan–Meier survival analysis to compare the survival outcomes. The high PFKFB3 expression group had significantly unfavorable overall survival (OS, *P* = 0.038), disease-specific survival (DSS, *P* = 0.04), and progress-free interval (PFI, *P* = 0.018, Fig. 7C). Next, we analyzed the ROC curves for assessing the diagnostic value of PFKFB3 in pRCC patients. The area under curve (AUC) of PFKFB3 was 0.765 (95%CI = 0.685–0.845), which supposed effective diagnostic value for pRCC patients. Furthermore, we explore time-depend ROC curves of OS, DSS, and PFI (Fig. 7D). Then, PPI network and GSVA algorithm were used to seek interacting proteins and potential pathways related to PFKFB3; the results showed that MYC Targets, mTORC1, and p53 pathways were enriched (Fig. 7E, F). Currently, tumor immune infiltrating cells are independent predictors of survival in cancers. We explored the underlying association between PFKFB3 and immune infiltration of cells. Our results revealed that PKFKB3 had a strong positive correlation with Treg cells, Type II IFN Response, and Th2 cells. For macrophages, however, there was a negative association with PFKFB3 (Fig. 7G). In addition, we investigated the association with some immune microenvironment molecules. Among them, PDCD1 (PD-1), CD274 (PD-L1), LAG-3, and CTLA-4 show a dramatically positive correlation with PFKFB3 (Fig. 7H).

### Silencing key gene PFKFB3 suppressed papillary renal cell carcinoma cells proliferation and migration

**Fig. 8:**
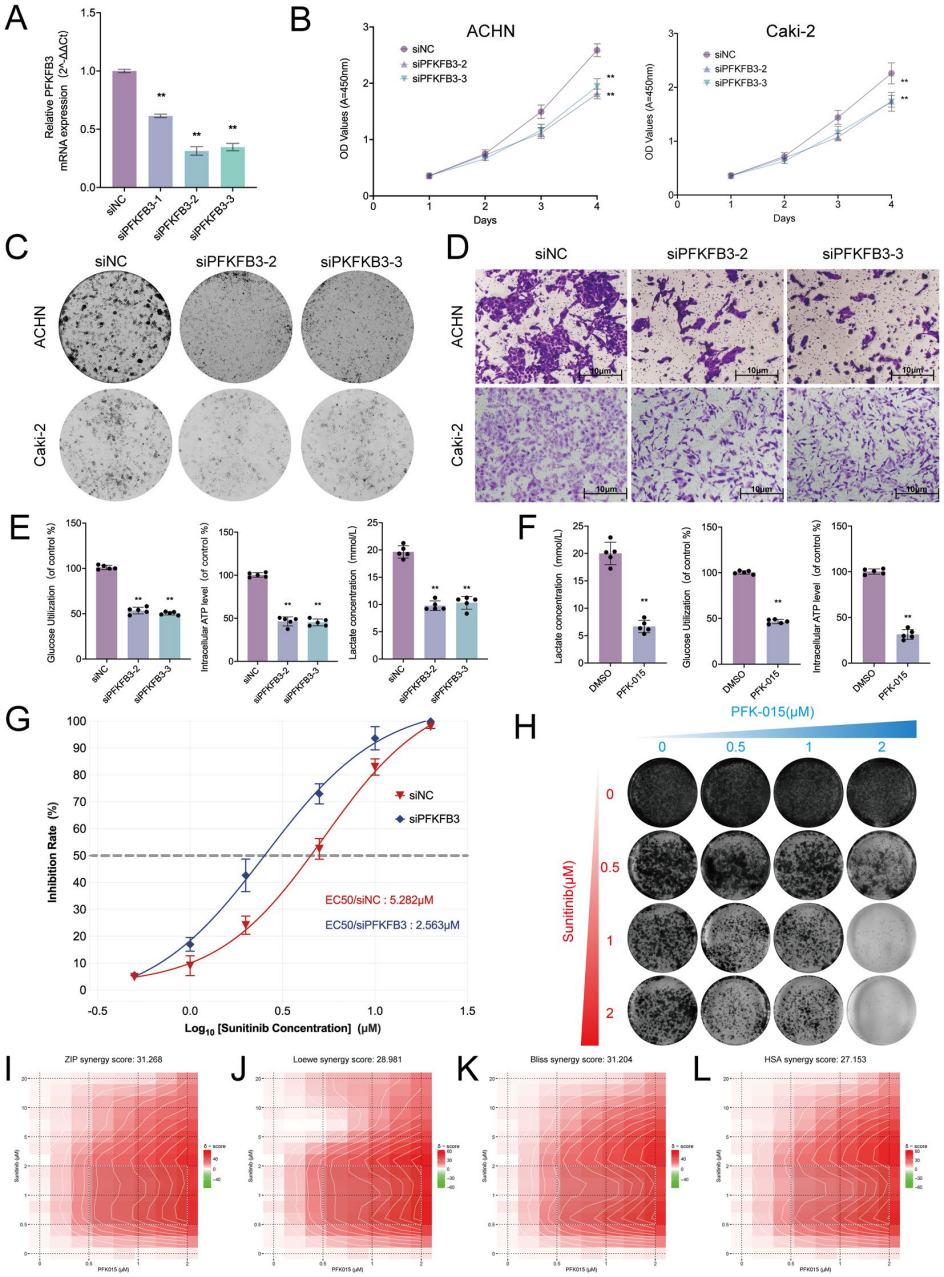
Silencing key gene PFKFB3 suppressed papillary renal cell carcinoma proliferation, migration and sunitinib resistance. **A** qRT-PCR to confirm the performance of siRNA targeting PFKFB3. **B** CCK-8 assay results indicated that PFKFB3 knockdown decreased cell proliferation. **C** Colony-formation efficiency of knockdown PFKFB3. **D** Transwell migration assay of knockdown PFKFB3 and control group. **E** Glucose uptake, lactate detection, and ATP detection assays of knockdown PFKFB3. **F** Glucose uptake, lactate detection, and ATP detection assays of ACHN cells at 1μM PFK-015. **G** Inhibition rate of ACHN siNC or siPFKFB3 cells treated with sunitinib. **H** The growth of ACHN cells after the treatment with sunitinib and/or PFK-015 for 10 days was determined using colony formation assay. **I**–**L** Synergy score plot based on ZIP (**I**); Loewe (**J**); Bliss (**K**) and HSA (**L**) models

To determine the biological function of PFKFB3, we transfected ACHN and Caki-2 papillary renal carcinoma cells with PFKFB3 small interfering RNA (siRNA), or control siRNA. PFKFB3 expression levels were measured by qRT-PCR to confirm the performance of siRNA (Fig. 8A). Consequently, we chose siPFKFB3-2 and siPFKFB3-3 for further experiments. Cell counting kit-8 (CCK-8) assay indicated that PFKFB3 knockdown decreased cell proliferation ability (Fig. 8B). Colony formation assay was also employed to determine the long-term impact of PFKFB3 on pRCC cell proliferation. We observed lower colony-formation efficiency among siPFKFB3-2 and siPFKFB3-3 groups than negative control group both in ACHN and Caki-2 cell lines after 10 days (Fig. 8C). Additionally, Transwell migration assay demonstrated that knockdown of PFKFB3 could decrease cells metastasis ability (Fig. 8D). Glucose uptake, lactate detection, and ATP detection assays revealed that PFKFB3 knockdown was able to significantly suppress glucose utilization, and lactate production and decrease intracellular ATP levels (Fig. 8E). Furthermore, PFK-015 (PFK15), a selective inhibitor of PFKFB3 was used for treatment in ACHN cells. Remarkably, we found PFK15 (1 μM) could reduce ACHN cell the level of glycolysis, consistent with the results of PFKFB3 depletion (Fig. 8F). In conclusion, the above results demonstrated that PFKFB3 may act as a positive regulator of tumor proliferation and migration in pRCC cells by promoting a glycolysis manner.

### PFK-015 and sunitinib synergistically inhibited pRCC cells proliferation

Nowadays, sunitinib is a first‐line recommended clinical treatment drug that targets multiple RTKs, such as VEGFR2 (Flk-1) and PDGFRβ [53, 54]. However, sunitinib resistance is still a major challenge for advanced renal cell carcinoma [55]. Emerging evidence has proved that glycolysis plays an important role in resistance to sunitinib [56, 57]. Since PFKFB3 was essential for pRCC glycolysis from the above analysis, we next investigated whether knockdown of PFKFB3 could re-sensitize pRCC to TKI inhibition. Importantly, we found that depletion of PFKFB3 could render pRCC cells sensitive to sunitinib (Fig. 8G). Given the poor response of pRCC patients to current therapies such as sunitinib, we speculated that the combined use of PFK-015 and sunitinib could be more active than a TKI inhibitor in pRCC cell lines [58, 59]. Surprisingly, by performing colony formation assays, we observed that PFK-015 and sunitinib showed highly synergistic effects on suppressing pRCC cell proliferation (Fig. 8H). Additionally, we calculated the synergy score for drug combinations and found a synergistic interaction between PFK-015 and sunitinib, with a high synergy score for ACHN cells [60, 61] (ZIP: 31.268; Loewe: 28.981; Bliss: 31.204; HSA:27.153; Fig. 8I–L).

## Discussion

PRCC is a second common subtype of renal cancer, with incidence accounting for 15% of total RCC cases approximately. Molecular characteristics have been well illustrated in ccRCC, but little is known in pRCC, especially for biomarkers in pRCC diagnosis, treatment and prognosis prediction. Metabolic reprogramming exists in most of tumors and fuels the rapid proliferation of cancer cells [62, 63]. As known, aerobic glycolysis has been widely studied to involve into cancer energy metabolism [13, 16] and affects tumor progression and therapeutic response, including renal cancer [16, 17, 64–67]. However, the key glycolysis-related regulators that participating pRCC biology and progression are undefined and still need further exploration. Herein, we applied integrative analysis by constructing glycolytic genes models in pRCC, and established its potential role in pRCC immune infiltration, clinical relevance and therapeutic strategies.

In the present study, we constructed a five glycolysis-related genes risk model in predicting pRCC patients’ survival and progression. Eleven glycose metabolic-related gene sets were primarily extracted and a total of 520 genes were identified for further analyzed. As known, cancer cells usually take up more nutrient and energy than normal cells for the maintenance of high proliferating rates, especially in renal cell carcinomas due to its specific metabolic mutations [4]. Emerging evidence suggests that the activation of specific metabolic pathway has a role in regulating angiogenesis and inflammatory signatures [68, 69]. Integrated multi-omics characterization has revealed different types of risk models based on glycolytic metabolism and validated their essential roles in determining RCC progression and therapeutic outcomes [15]. It has been reported that a glycolysis-related Long noncoding signature participated renal cancer evolution and released potential abilities in deciding RCC patients’ prognosis. Zhang et al. collected CNV, SNV, and mRNA expression from TCGA ccRCC cohort and established a predictive model consisting of 13 glycolysis-correlated genes. This predictive model including a batch of classical or novel glycolytic genes, such as RPIA, G6PD, PSAT1, ENO2, HK3, IDH1, PDK4, PGM2, PGK1, FBP1, OGDH, SUCLA2, and SUCLG2 [65]. Using the same dataset, Wang et al. screened differentially expressed genes related to glucose metabolism pathway, finally selected six glucose metabolism-related DEGs (FBP1, GYG2, KAT2A, LGALS1, PFKP, and RGN) and developed a risk signature [66]. Moreover, a four glycolysis-relevant signature (B3GAT3, CENPA, AGL and ALDH3A2) was also built and applied to better predict ccRCC patients [70]. To our knowledge, pRCC is one of the most immune-infiltrated tumors [71–73]. Features of the tumor microenvironment heavily affect disease biology and may affect responses to systemic therapy [74–78]. It has also been announced that a novel glycolytic risk signature could not only participate in RCC progression and prognosis but also affect the immune microenvironment of RCC. It included CD44, PLOD2, KIF20A, IDUA, PLOD1, HMMR, DEPDC1, and ANKZF1 in the model, which might exert the possibility of being a biomarker for the immunotherapy response [79]. However, little attention has been concentrated on the relationship between glycolytic genes and pRCC progression.

Our study comprehensively filtered DEGs using various glycolytic-related gene sets, WGCNA, and Immune infiltration landscape, and firstly identified 34 overlapping genes as a primary glycolysis-immune signature in pRCC. GO enrichment analysis declared that these 34 key genes might be strongly correlated with carbohydrate metabolic process, hexose metabolic process, and glucose metabolic process, and KEGG pathway analysis also showed potential participating pathways, including Insulin signaling pathway, HIF-1 signaling pathway, and AMPK signaling pathway. Nine candidate genes (PPP4R3B, LDHA, PPIB, YWHAZ, NUP107, PFKFB3, FOXK2, CRTC2, and NUP43) were further screened out, and LASSO Cox Regression algorithm and multivariate Cox analysis indicated the most powerful genes and conducted a five genes GIRPI. Previously reported, researchers have established several predicting models from glycolysis-relevant genes, which showed robust function in demonstrating renal cancer outcomes and progression [5, 80]. Different risk models might represent similar predictive roles in tumor survival decision-making. However, the data collection and extracted approach between different researchers would bring discrepant results. Currently, there is no standard screening route for bioinformatic analysis, which limited the reliability of risk models in guiding patients’ outcomes. Therefore, our present research used different analyzing approaches including TCGA datasets, WGCNA, immune and glycolysis clusters, then combined with integrated Cox regression analysis, and finally built a five-gene-based prediction signature. This signature certainly represented a powerful effect in evaluating the predictive value of training and testing datasets. In addition, we identified several potential therapeutic strategies for pRCC based on our risk signature, which provided more treatment opportunities for pRCC patients even with unresectable cancer mass or late-stage tumors.

Considering the unique role of individual genes, we further filtered the highest differentially expression gene from GIRPI and evaluated the expression difference in renal cancer cell lines. A glycolytic-related gene called PFKFB3 was finally screened out for subsequent verification. During the past several years, the function of PFKFB3 in cancer has advanced considerably. Knowledge of the vital role of PFKFB3 in regulating glycolysis, accumulating evidence has demonstrated that PFKFB3 participated in multiple cancer events, including carcinogenesis, cancer cell proliferation, vessel aggressiveness, drug resistance, and tumor microenvironment [81]. In view of renal cancer as a metabolic disease [4], PFKFB3 mediated glycolytic pathways should affect RCC development and progression. However, the regulating role of PFKFB3 in RCC glycolysis metabolism is rarely elucidated currently, much less in pRCC. Our study primarily demonstrated the abnormal expression profile of PFKFB3 in pRCC. Experimental assays further verified that PFKFB3 could promote renal cancer cell proliferation and migration in vitro, confirming its oncogenic potential in tumor progression.

As accumulates of PFKFB3 in cancer metabolism, researchers have aroused increasing interest in developing inhibitors targeting PFKFB3 for anti-neoplastic therapy [82]. Currently, potent and selective inhibitors of PFKFB3, such as PFK-015 and PFK-158 have been identified and undergone clinical trials for treating late-stage patients with cancer [83–85]. We also found that PFK-015 exerted a substantially suppressive role in RCC cell proliferation in vitro, showing a similar function to a specific glycolysis inhibitor 2-DG. These results indicated the feasibility of PFKFB3 as a potential target of pRCC intervention therapy, which provided a more extensive choice for treating RCC patients. On the other hand, accumulating resistance to targeted therapy would also allow for broader alternatives including drug combinations. Furthermore, we confirmed that PFK-015 and sunitinib could highly synergistically suppress pRCC cell proliferation, which may provide a promising new treatment strategy for advanced renal cell carcinoma making this combination drug therapy. The discovery of biomarkers that predict benefit and the use of a suitable tolerance combination are crucial pillars in improving renal cell cancer prognosis [86, 87].

## Conclusion

In this study, we constructed a Glycolysis-Immune Related Prognostic Index and performed comprehensive analyses to elucidate tumor immune infiltration, clinical relevance, and therapeutic targets of pRCC using bioinformatic methodology. GIRPI is closely associated with pRCC prognosis, progression, immune infiltration, and therapeutic response. PFKFB3, as a vital regulator of glycolysis and mediates sunitinib resistance, was finally screened out as a promising glycolytic biomarker in pRCC.

### Supplementary Information

**Additional file 1:Figure S1**. Identification of differently expressed genes in the TCGA-KIRP dataset; **Figure S2**. Tumor microenvironment immune infiltration landscape in pRCC subtypes; **Figure S3**. Construction of GIRPI model; **Figure S4**. Kaplan-Meier curves depicted the survival difference of GIRPI genes; **Figure S5**. Genetic alterations of five signature genes; **Figure S6**. Distribution of GIRPI genes in PRCC tissues obtained by UMAP algorithm; **Table S1**. Oligonucleotide sequences used in this research.

## Data Availability

The datasets generated during the current study are available in the TCGA and GEO database (TCGA-KIRP cohort, GSE152938, and GSE131685). Other data that support the findings of this study are available from the corresponding author upon reasonable request.
