# Supplementary material for: Multi-omics and immunogenomics analysis revealed PFKFB3 as a targetable hallmark and mediates sunitinib resistance in papillary renal cell carcinoma: in silico study with laboratory verification
Source: Eur J Med Res. 2024 Apr 15;29:236. doi: 10.1186/s40001-024-01808-5 (PMC11017615; doi:10.1186/s40001-024-01808-5)
Supplement: Supplementary file 1 — Additional file 1:Figure S1. Identification of differently expressed genes in the TCGA-KIRP dataset; Figure S2. Tumor microenvironment immune infiltration landscape in pRCC subtypes; Figure S3. Construction of GIRPI model; Figure S4. Kaplan-Meier curves depicted the survival difference of GIRPI genes; Figure S5. Genetic alterations of five signature genes; Figure S6. Distribution of GIRPI genes in PRCC tissues obtained by UMAP algorithm; Table S1. Oligonucleotide sequences used in this research. [file 40001_2024_1808_MOESM1_ESM.docx]

**Supplementary materials:**

**Figure S1: Identification of differently expressed genes in the TCGA-KIRP dataset.**

(A). Volcano plot showed DEGs identification from TCGA-KIRP cohort. (B) Heatmap of top 100 up- and downregulated genes. (C). Analysis of the scale-free fit index (left) and the mean connectivity (right) for various soft-thresholding powers. (D). Scatter plot of gene significance in the turquoise module. (E). The CDF plots of two distinct immune subtypes. (F). The CDF plots of two distinct subclasses mediated by glycolysis-related genes.


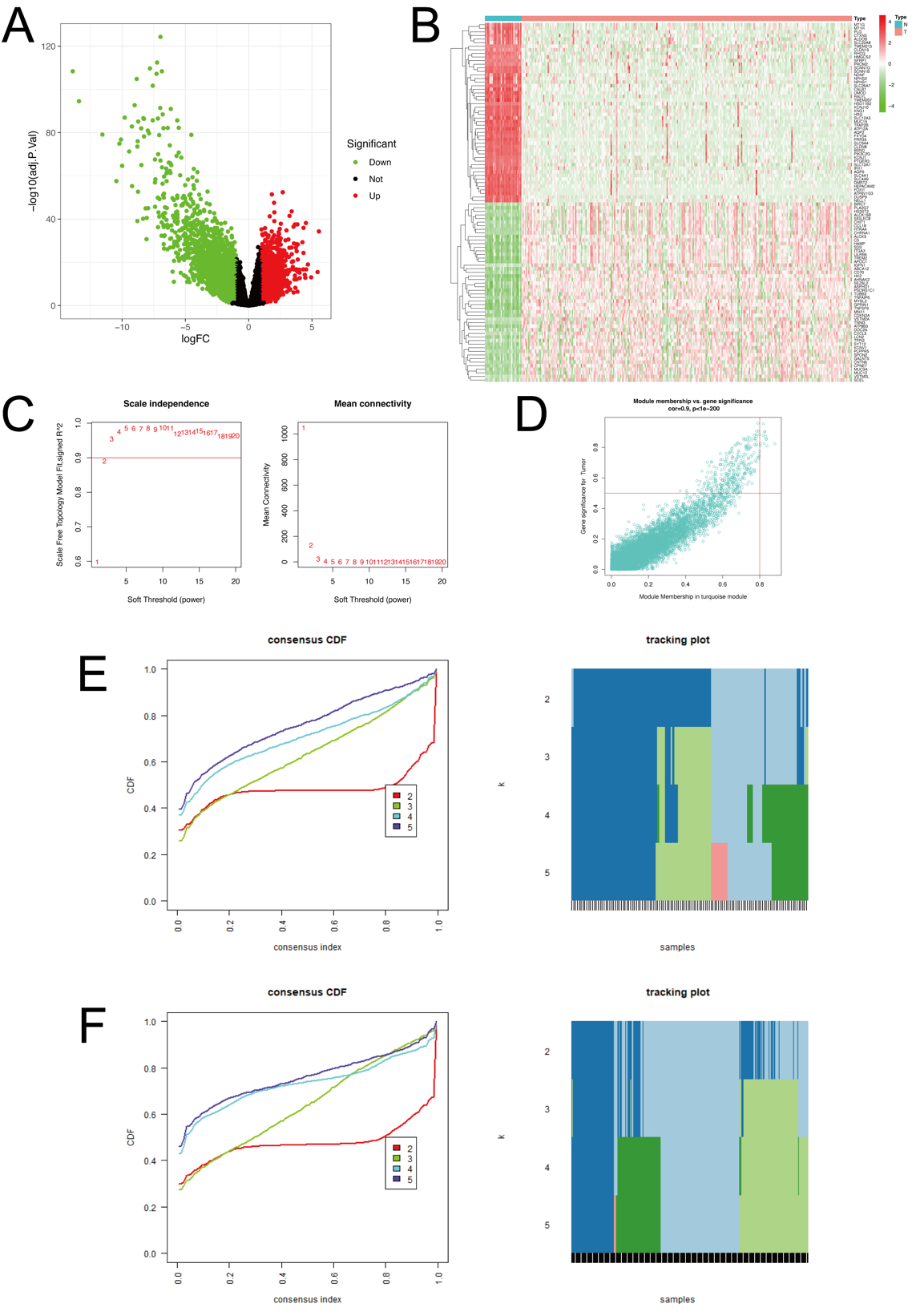


**Figure S2: Tumor microenvironment immune infiltration landscape in pRCC subtypes.**

(A). The association between different immune cell subsets and pRCC clinical information.

(B). GSVA enrichment analysis shows the activation states of biological pathways in distinct immune clusters. * *P* < 0.05; ** *P* < 0.01; *** *P* < 0.001. ns, not significant.


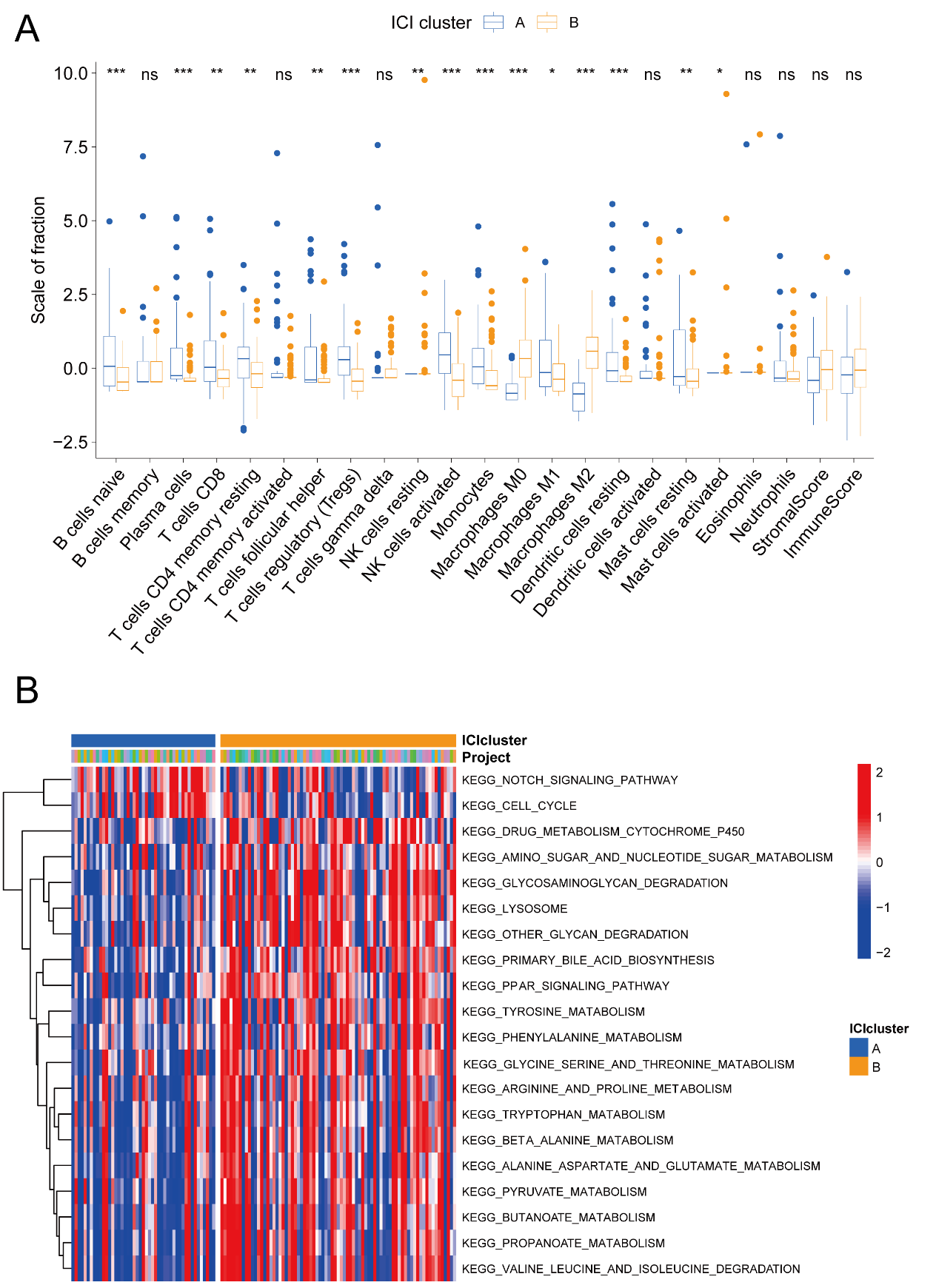


**Figure S3: Construction of GIRPI model.**

(A). Filter out nine candidate genes through univariate Cox regression. (B). The LASSO Cox regression model was constructed from 34 WGCNA-glycolysis-immune-related overlapping genes. (C). Distribution of LASSO coefficients of GIRPI risk signature. (D). Heatmap revealed the landscape of corresponding clinicopathological features and the expression level of five selected genes.


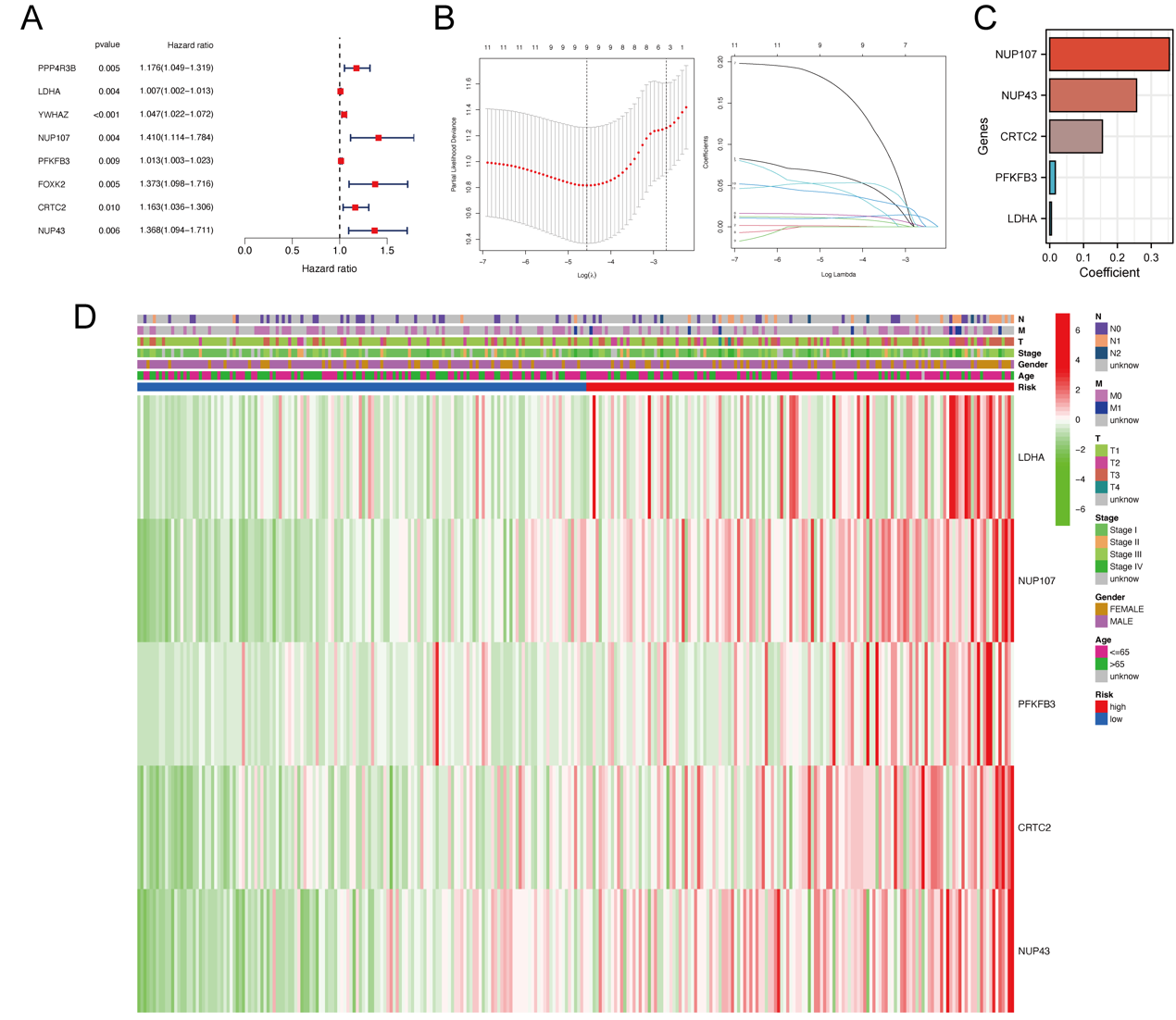


**Figure S4: Kaplan-Meier curves depicted the survival difference of GIRPI genes.**

(A). Association between CRTC2, NUP43, NUP107 and LDHA expression and overall survival time in the TCGA-KIRP dataset. (B). Association between CRTC2, NUP43, NUP107 and LDHA expression and disease-free survival time in the TCGA-KIRP dataset.


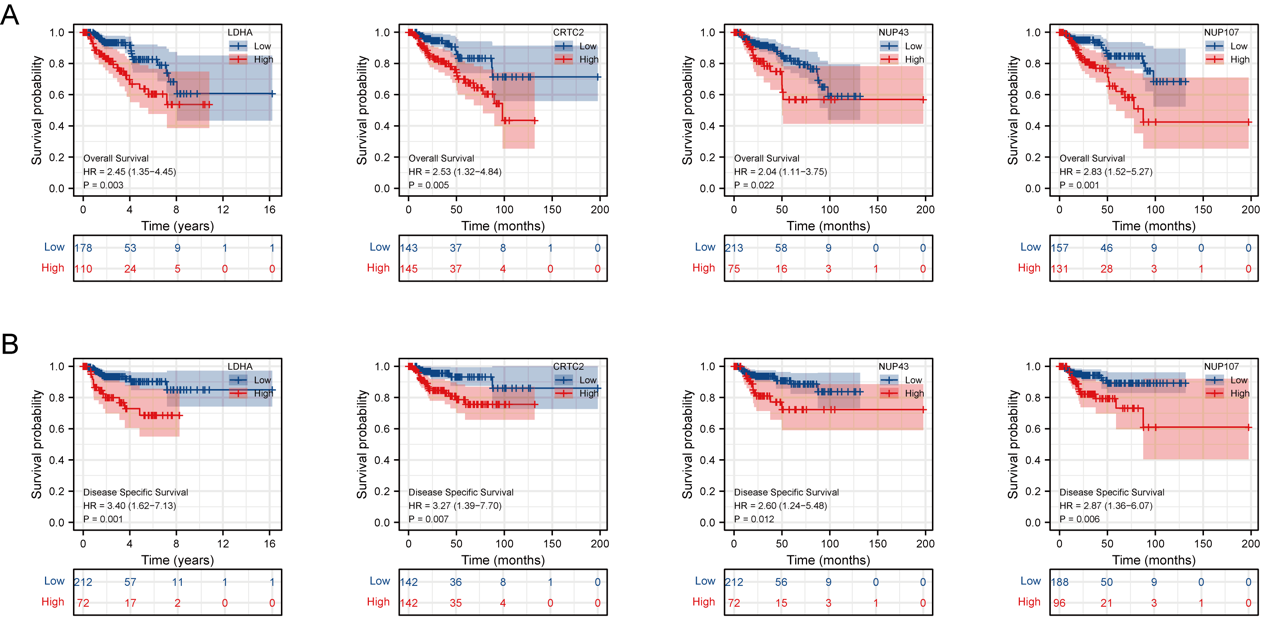


**Figure S5: Genetic alterations of five signature genes.**

(A). Genetic alteration information of five signature genes was explored by querying cBioPortal database. (B). Association of five signature genes’ copy number variation with immune infiltration.


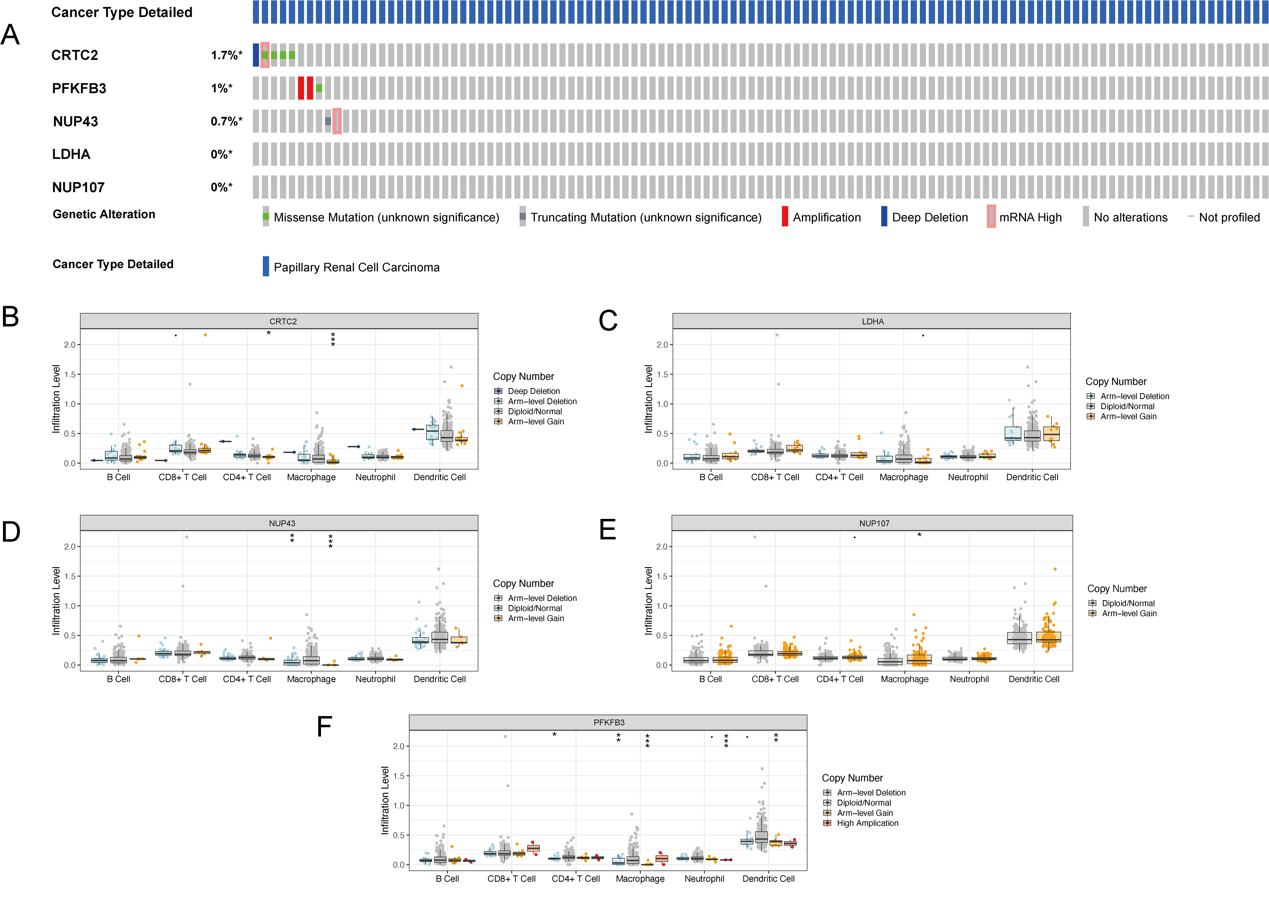


**Figure S6: Distribution of GIRPI genes in pRCC tissues obtained by UMAP algorithm.
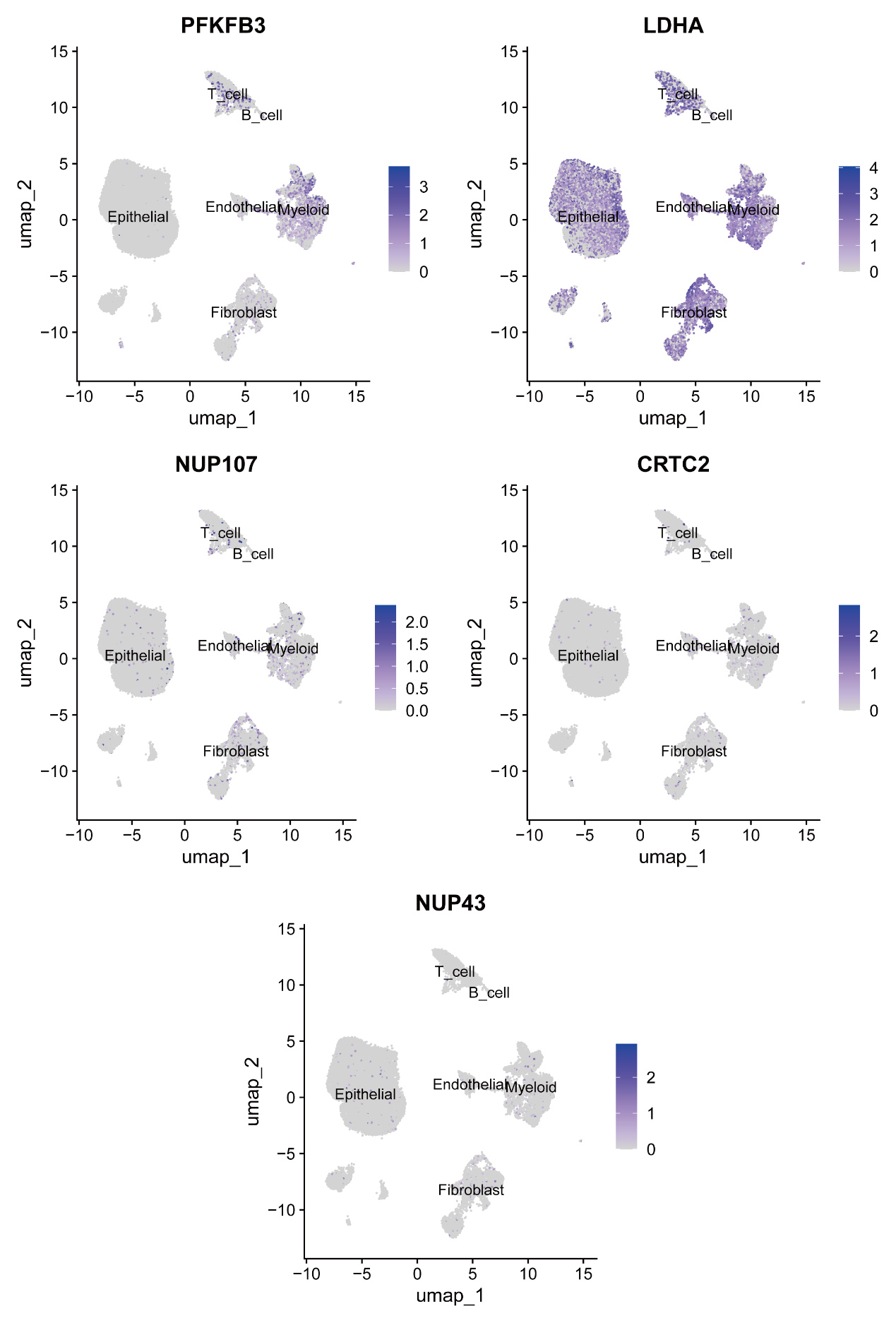
**

**Table S1 Oligonucleotide sequences used in this research**

| Primers | Sequences | |
| --- | --- | --- |
| PFKFB3 | Forward | TTGGCGTCCCCACAAAAGT |
|  | Reverse | AGTTGTAGGAGCTGTACTGCTT |
| CRTC2 | Forward | CCGGTTACAGGCCCAAAAACT |
|  | Reverse | AATGTGGCGGGTGTATCGG |
| NUP107 | Forward | CACGGACTGCACGGAAACA |
|  | Reverse | GAGTTCGAGGGATAACCTGGT |
| NUP43 | Forward | TGCCTCCGGGAAGTTTACAGA |
|  | Reverse | TCTCCTTCAAACCCTCCATCA |
| LDHA | Forward | ATGGCAACTCTAAAGGATCAGC |
|  | Reverse | CCAACCCCAACAACTGTAATCT |
| β-actin | Forward | ATGACTTAGTTGCGTTACACC |
|  | Reverse | GACTTCCTGTAACAACGCATC |
| siRNA-NC | Forward | UUCUCCGAACGUGUCACGUTT |
|  | Reverse | ACGUGACACGUUCGGAGAATT |
| siRNA-PFKFB3-1 | Forward | GCAAGACCUACAUCUCCAATT |
|  | Reverse | UUGGAGAUGUAGGUCUUGCTT |
| siRNA-PFKFB3-2 | Forward | CCAAUAUCAUGGAAGUUAATT |
|  | Reverse | UUAACUUCCAUGAUAUUGGTT |
| siRNA-PFKFB3-3 | Forward | GGUGUGCGACGACCCUACATT |
|  | Reverse | UGUAGGGUCGUCGCACACCTT |
